# Bromodomain Inhibition Attenuates the Progression and Sensitizes the Chemosensitivity of Osteosarcoma by Repressing GP130/STAT3 Signaling

**DOI:** 10.3389/fonc.2021.642134

**Published:** 2021-06-08

**Authors:** Yafei Jiang, Gangyang Wang, Haoran Mu, Xiaojun Ma, Zhuoying Wang, Yu Lv, Tao Zhang, Jing Xu, Jinzeng Wang, Yunqi Li, Jing Han, Mengkai Yang, Zongyi Wang, Ke Zeng, Xinmeng Jin, Song Xue, Mingzhu Yin, Wei Sun, Yingqi Hua, Zhengdong Cai

**Affiliations:** ^1^ Department of Orthopaedics, Shanghai General Hospital, Shanghai Jiao Tong University School of Medicine, Shanghai, China; ^2^ Shanghai Bone Tumor Institution, Shanghai, China; ^3^ National Research Center for Translational Medicine, Ruijin Hospital Affiliated to Shanghai Jiao Tong University (SJTU) School of Medicine, Shanghai, China; ^4^ Department of Dermatology, Hunan Engineering Research Center of Skin Health and Disease, Hunan Key Laboratory of Skin Cancer and Psoriasis, Xiangya Hospital, Central South University, Changsha, China

**Keywords:** osteosarcoma, BRD4, NHWD-870, chemosensitivity, PDX 3

## Abstract

Osteosarcoma is the most common primary malignant bone tumor, and there are few ideal clinically available drugs. The bromodomain and extraterminal domain (BET) protein is an emerging target for aggressive cancer, but therapies targeting the BET in osteosarcoma have been unsuccessful in clinical trials to date, and further exploration of specific BET inhibitors is of great significance. In our study, we demonstrated that NHWD-870, a potent BET inhibitor in a phase I clinical trial, significantly inhibited tumor proliferation and promoted cell apoptosis by reversing the oncogenic signature in osteosarcoma. More importantly, we identified NHWD-870 impeded binding of BRD4 to the promoter of GP130 leading to diminished activation of JAK/STAT3 signaling pathway. Furthermore, GP130 knockdown significantly sensitizes the chemosensitivity *in vitro*. In OS cell-derived xenografts, NHWD-870 effectively inhibited the growth of osteosarcoma. Beyond that, NHWD-870 effectively inhibited the differentiation and maturation of precursor osteoclasts *in vitro* and attenuated osteoclast-mediated bone loss *in vivo*. Finally, we confirmed the efficacy of synthetic lethal effects of NHWD-870 and cisplatin in antagonizing osteosarcoma in a preclinical PDX model. Taken together, these findings demonstrate that NHWD-870, as an effective BET inhibitor, may be a potential candidate for osteosarcoma intervention linked to its STAT3 signaling inhibitory activity. In addition, NHWD-870 appears to be a promising therapeutic strategy for bone-associated tumors, as it interferes with the vicious cycle of tumor progression and bone destruction.

## Introduction

Osteosarcoma (OS) is one of the most aggressive tumors of the bone among children and young adolescents. Osteosarcoma usually occurs in the metaphysis of long bones, most commonly in the distal femur (43%), proximal tibia (23%), and humerus (10%) (1). Although the overall survival of patients with osteosarcoma has improved to a five-year survival of 70%, the outcomes of patients with metastatic or recurrent OS remain poor (2). Several key oncogenic genes and pathways have been identified in OS, including the NOTCH, WNT/beta-catenin, and JAK/STAT3 signaling pathways as well as the transcription factors RUNX2 and Osterix, while the molecular mechanisms of osteosarcoma genesis and progression remained poorly understood.

Bromodomain and extraterminal domain (BET) proteins, as the most famous epigenetic readers, regulate gene expression and are involved in various cancer pathogenesis (3, 4). The significant clinical value of BET targeted therapy has been proven, and several BET inhibitors have been designed and applied (5–7). In our previous study, we reported the discovery and characterization of the novel BET inhibitor NHWD-870, and mechanism by which BRD4 inhibition suppresses tumor growth (8, 9). The present study aimed to further explore the effects of NHWD-870 in osteosarcoma and the related mechanism. This study is expected to further accelerate the development of small-molecule inhibitors targeting BET and provide new drug options for epigenetic-driven tumors, including osteosarcoma.

## Material and Methods

### Cell Culture and the Regents

Human osteosarcoma cell lines (143B, HOS, U2OS, U2OSR and SJSA1) were purchased from American Type Culture Collection (ATCC, Manassas, VA). Cells were maintained in Dulbecco modified Eagle medium (DMEM)/High Glucose medium (Hyclone, Logan, UT) supplemented with 10% fetal bovine serum (FBS; Sigma-Aldrich, St Louis, MO), streptomycin (100 mg/mL), and penicillin (100 U/mL). Cultures were incubated at 37°C with 5% CO_2_ humidified incubator. NHWD-870 was synthesized by Ningbo Wenda Pharma (Ninghai, Zhejiang, China), and the synthesis procedure were described (**Supplementary Figure 1**).

### Cell Viability Assay

Cell viability at 24 and 48 after NHWD-870 administration was analyzed using cell counting assay Kit-8 (CCK-8; Dojindo, Japan) according to the manufacturer’s protocol. Cells were incubated in 10% CCK-8 diluted in normal culture media at 37 °C for 1 hour. The absorbance of each well was measured with a microplate reader set at 450 nm.

### Colony Formation

Cells at a density of 1000 cells per well were plated into a 6-well plate and incubated at 37°C with 5% CO_2_ humidified incubator for 7 days. The culture medium was changed every 3 days. Then, cells were washed with phosphate buffered saline (PBS) and fixed with 4% cold paraformaldehyde (PFA), after stained with 1% crystal violet (Sigma-Aldrich), the colonies in each well were counted. Three replicate wells were included for each group.

### Cell Apoptosis Assay

Flow cytometry was performed for cell apoptosis using a cell apoptosis detection kit (BD Biosciences, Franklin Lakes, NJ, USA) according to the manufacturer’s protocol. 143B and HOS cells were seeded into 6-well plates at density of 1*10^5^ cells/well. After 24 hours NHWD-870 administration, the cells were harvested by trypsinization and washed with PBS. Then, cells were resuspended in Annexin-binding buffer, and 5 μl Annexin V-FITC plus 1 μl PI were appended for double-staining. The apoptotic cells were performed using flow cytometric analyzes (FACS Calibur, BD Biosciences) and the Flowjo software (Tree Star Corp, Ashland, USA) was used to analyze the results.

### Immunofluorescence Assay

After treatment, 143B cells were fixed with 4% paraformaldehyde (PFA) for 15 minutes at room temperature. After permeabilized with 0.1% Triton X‐100 for 15 minutes, the cells were blocked in 0.1% bovine serum albumin (BSA) for 30 minutes and then incubated with STAT3 antibody at 4°C overnight. After washing 3 times with a TBS‐T solution, Alexa Fluor 594‐conjugated secondary antibodies (1:400) were incubated for 1 hour at room temperature. Cell nuclei were stained with DAPI (Solarbio, 1:200) for 3 minutes. After washing 3 times with PBS, the images were captured under confocal microscopy.

### Western Blot

After treatment, total protein was extracted from cells using cold ripa protein lysis buffer. The protein concentrations of the lysates were measured by the Protein BCA Assay Kit (Bio-Rad, Hercules, CA, USA). 20 μg of protein was loaded for western blot assay, separated with 10% SDS-PAGE and transferred to a polyvinylidene fluoride (PVDF) membrane. After blocked with 5% milk for 1 h at room temperature, the membrane was incubated at 4°C overnight with primary antibodies, and then, the membrane was washed three times for 10 min with TBS-T solution and incubated for 1 h with the corresponding HRP-conjugated secondary antibodies (1:5000, Abgent). Chemiluminescent detection was performed using an ECL kit (Pierce Chemical, Rockford, IL, USA) and Bio-Rad ChemiDoc MP Imaging System. All experiments were performed in triplicate.

### *In Vitro* Osteoclastogenesis Assay

For the *In Vitro* osteoclastogenesis assay, bone marrow cells (BMMs) were isolated from the femurs and tibias of C57BL/6 mice and cultured in α-MEM supplemented with 10% FBS and macrophage colony stimulating factor (M-CSF) (10 ng/mL) for 24 hours; the BMMs were allowed to adhere overnight and then cultured with mM-CSF (30 ng/mL) and Receptor Activator for Nuclear Factor-κ B Ligand (RANKL) (100 ng/mL) in 96-well plates in the presence or absence of NHWD-870 for 7 days with media changed every 2 days. After treatment, cells were fixed with 4% paraformaldehyde (PFA) for 15 minutes at room temperature. After permeabilized with 0.1% Triton X-100 for 10 min, the cell were incubated with Phalloidin-Fluor 594 reagent for 1 hour at room temperature, after washed with PBS for three times, nuclei were counterstained with DAPI for 3 min. Fluorescence images were captured under fluorescence microscopy.

### RNA Sequencing and Date Processing

The RNA sequencing was performed by Meiji Biotech (Shanghai, China). Briefly, 143B and HOS OS cells treated with DMSO or NHWD-870 (1 μM) were collected with biological duplicates. The cells were lysed for RNA extraction with TRIzol reagent following the manufacturer’s protocol. Samples with an RNA Integrity Number (RIN) ≥ 8 were used for the subsequent libraries construction and sequencing. The raw data was processed as described previously. We defined the significantly differentially expressed genes between DMSO group and NHWD-870 treated group (Log2FC>1, FDR ≤ 0.01). The function and pathways enrichment of DEGs were analyzed using DAVID database.

### SiRNA Mediated Knockdown

143B cells were seeded in a six well plate 24 h before transfection. siRNA duplex targeting GP130 were transfected using Lipofectamine 2000 (Invitrogen Life Technologies) according to the manufacturer’s protocols. The sequences of the GP130 and scramble control were as follows:

GP130: 5’-CAGAATTGTTTATGGAATCACA-3’.
Scramble: 5’-AATTCTCCGAACGTGTCACGT-3’.

### Chromatin Immunoprecipitation (ChIP) Assays

ChIP assay was performed using an SimpleChIP Enzymatic ChIP kit (Cell Signaling Technology) according to manufacturer’s protocol. Immunoprecipitated DNA was analyzed by real-time PCR and the data was expressed as fold enrichment (2E (-ΔΔCt (ChIP/IgG control)) and normalized to the input (DMSO group). The primer and probe specific for BRD4 binding sites were as fellow:

Forward primer: 5’-GCGCGAGTTCCTCAAATGTT-3’
Reverse primer: 5’-TCCCACTCGCACATGACTCA-3’
Probe: CCTGCGTTGCCAGGACCGTCC.

### Tumor Xenografts

We established a mouse osteosarcoma model to evaluate the effects of NHWD-870 on osteosarcoma progression. After anesthesia, 10 μl of 143B cell suspension (1 × 10^6^ cells/ml) was injected into the marrow space of the proximal tibial with a 27-gauge needle. After injection, the mice were randomly divided into four groups: sham DMSO control (n = 6); 143B with DMSO (vehicle, n = 6) and 143B with low NHWD-870 (1 mg/kg, n = 6) and high NHWD-870 (2 mg/kg, n = 6) doses. Mice in the low and high NHWD-870 groups were given intragastric administration with NHWD-870 every day for 3 weeks. Tumor volume was measured at 1-week intervals until the animal was sacrificed and was calculated by the following equation: volume = length×width^2^/2. After sacrifice, the tibia was fixed in 4% paraformaldehyde for micro-computed tomography (micro-CT) and histological analyses. Additionally, the liver and kidney were fixed for hematoxylin and eosin (H&E) staining to assess the toxic effects of NHWD-870. The experimental protocol was approved by the Animal Ethics Committee of Shanghai General Hospital.

### Patient-Derived Xenograft Model

4-week-old female BALB/C‐nu mice (Shanghai SLAC Laboratory Animal Co., LTD., Shanghai, China), raised in the standard animal laboratory at Shanghai General Hospital (21°C, 55% humidity, 12h light and dark cycle).

As our earlier reported, the PDX model was established using surgical specimens from osteosarcoma patients (10). The specimen is cut into 2×2×2 mm^3^ sections in a tissue culture medium under aseptic conditions. The tumor was then subcutaneously implanted into the BALB/C‐nu mice. After successful established, tumor tissue samples were passed through nude mice to prepare for follow-up studies. After 2 weeks, DMSO, NHWD-870 (2 mg/kg/day) and/or Cisplatin (3 mg/kg/week) were given intragastric administration. Mice injected with DMSO were used as control. Tumor volume was measured every 3 days. After 4 weeks of treatment, all mice were killed and tumor tissue was collected for subsequent histological testing.

### Immunohistochemical Histopathology Assessment

Formalin‐fixed tissue samples of osteosarcoma patients (from tissue micro-array) and xenograft tumor (in animal experiments) were used for histological assessment. The tumor and important organs, including heart, lung, liver and kidney sections, were stained with H&E, and the tumor specimens were immunostained with PCNA (CST, 13110, 1:5000) and p-STAT3 (CST, 9145, 1:200) and GP130 (Abcam, ab202850, 1:400). Images were captured using microscope (Leica).

### TUNNEL Staining

To detect apoptotic cells in tumor tissue, sections were stained with terminal deoxyribonucleoside transferase DUTP-labeled terminal marker (TUNEL) according to manufacturer’s instructions. The nucleus was stained with DAPI. The sections were sealed with anti-fluorescence quenching agent and detected under fluorescence microscope.

### Micro CT Analysis

After treatment, hind limbs of mice were dissected and fixed with 4% PFA for 24 hours, After fixation, the micro structures of limbs were analyzed with micro CT. The axial scan was performed using the high resolution cathode CT (70 kV, 200 200 μA) in YUEBO Company (Hangzhou, China). The tibia was defined as an area of interest (ROI). The bone trabeculae were analyzed using the software provided by the manufacturer, including bone density (BMD), bone volume/total tissue volume (BV/TV), number of bone trabeculae (Tb.N), thickness of bone trabeculae (Tb.Th), separation of bone trabeculae (Tb.Sp), and structural model index (SMI).

### Osteoclast TRAP Staining

After μCT analysis, the fixed bone tumor samples were embedded in paraffin blocks for sectioning into 4 μm sections, and were stained with H&E and TRAP activity. TRAP-positive multinucleated cells with more than 3 nuclei were counted as osteoclasts and their cell spread area was calculated.

### Systemic Toxicity Test

After drug treatment, the important organs, including heart, liver, spleen, lung and kidney, were collected and fixed with 4% PFA, H&E staining was performed to evaluate the systemic toxicity of NHWD-870 on mice.

### Statistical Analysis

All statistical analyses were performed using SPSS 25.0 software (IBM Corporation, New York, USA). Data in this study are presented as mean ± SEM or representative images of at least three independent experiments. Differences were analyzed with the Student’s t-test or one-way analysis of variance (ANOVA), and significance was set at P < 0.05; *, P < 0.05; **, P < 0.01; ***, P < 0.001, and ****, P < 0.0001, respectively.

## Results

### NHWD-870 Inhibits Proliferation and Induces Apoptosis of Osteosarcoma *In Vitro*

**Figure 1:**
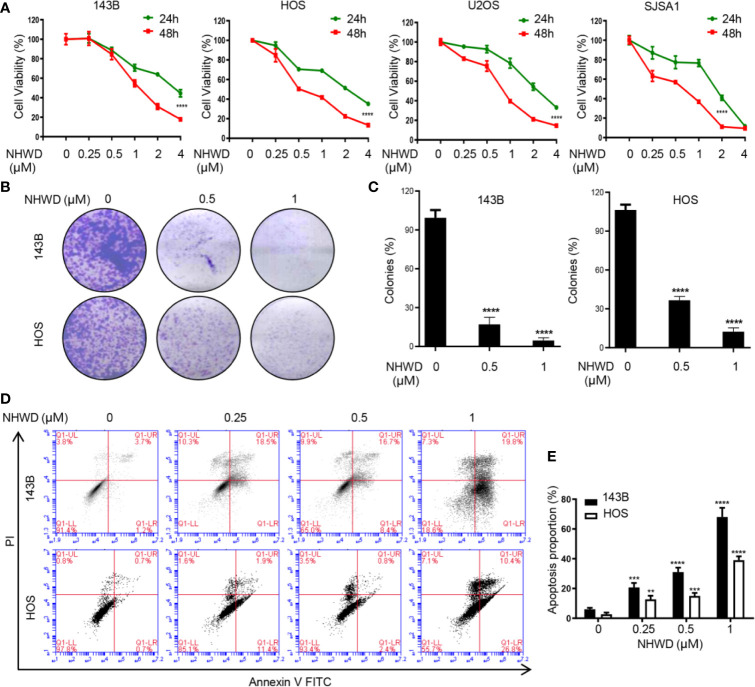
NHWD-870 inhibits the proliferation and induces the apoptosis of human osteosarcoma cells *in vitro*. **(A)** 143B, HOS, U2OS, and SJSA1 cells were seeded at 5000 cells per well and treated with NHWD-870 at the indicated concentration for 24 or 48 hours. Cell activity was measured by CCK8 . (****P < 0.0001). **(B)** Colony formation was used to evaluate the inhibitory effects of NHWD-870 on osteosarcoma cell proliferation. **(C)** The statistical results of the osteosarcoma colony formation test showed that NHWD-870 significantly reduced the colony formation ability of osteosarcoma cells . (****P < 0.0001). **(D)** Cell apoptosis was analyzed using Annexin V‐FITC/PI flow cytometry. After treatment with the indicated concentration of NHWD-870 for 24 h, Annexin V‐FITC/PI staining was used for flow cytometry analysis of cell apoptosis. Data analysis was performed using Flow Jo software. **(E)** The FACS statistics and the percentages of apoptotic cells in the indicated cell populations are shown in the histogram and expressed as the mean ± SD of three independent experiments. (**P < 0.01; and ***P < 0.001, and ****P < 0.0001).

As our previous reported that the potent BRD4 inhibitor NHWD-870 strongly suppresses the growth of multiple solid tumors by depleting phosphorylated BRD4 and c-MYC (8). To further explore the effects of NHWD-870 in osteosarcoma, human osteosarcoma cell lines 143B, SJSA1, U2OS and HOS were incubated with NHWD-870 at the indicated concentrations for 24 and 48 hours, cell viability was evaluated by CCK8 analysis. As shown in **Figure 1A**, NHWD-870 inhibited osteosarcoma cell proliferation in a dose-dependent and time-dependent manner. Furthermore, clone formation assay further indicated that NHWD-870 significantly reduced the clone number compared to that of the control group (**Figures 1B, C**). Flow cytometry indicated that significant apoptosis occurred in osteosarcoma cells after incubation with NHWD-870 for 24 hours, which was positively correlated with the concentration of NHWD-870 (**Figures 1D, E**). The results suggest that the antitumor effects of NHWD-870 were exercised by promoting cell apoptosis.

### NHWD-870 Reverses Oncogenic Signature in OS

**Figure 2:**
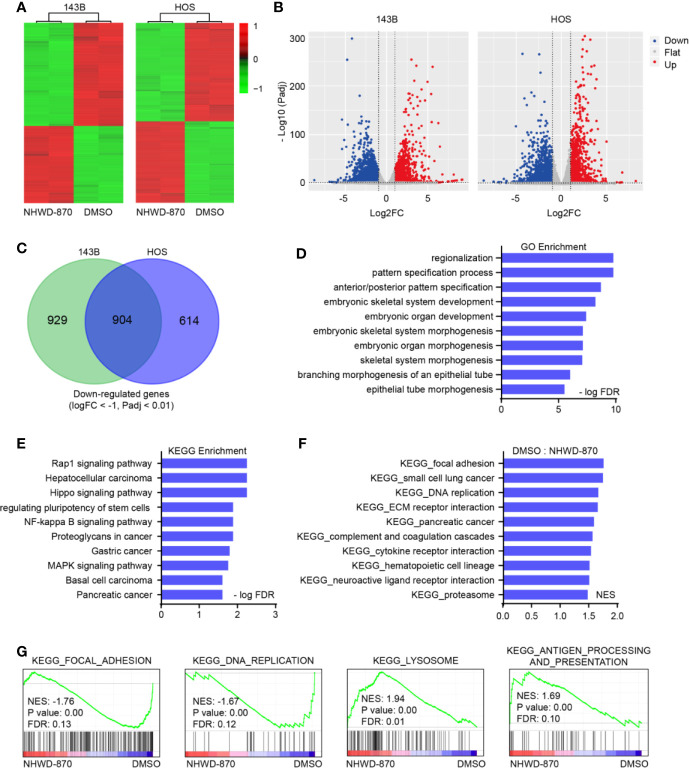
NHWD-870 reverses oncogenic signature in osteosarcoma. **(A)** Heatmap of differentially expressed genes (log2FC<-1, log2FC<-1, and adjusted p value < 0.01) in 143B and HOS cells treated with NHWD-870 (1 μM for 24 hours) versus DMSO. **(B)** Volcano plot showing the differentially expressed genes (DEGs) between the NHWD-870-treated and DMSO groups. (adjusted p value < 0.01). **(C)** Venn diagram of the significantly downregulated transcripts in 143B and HOS osteosarcoma cells treated with 1 μM NHWD-870 for 24 hours. **(D)** GO analyses of the 904 overlapping significantly downregulated genes. The top 10 hits in each category were presented. **(E)** KEGG analyses of the 904 overlapping significantly downregulated genes. Top 10 hits in each category were presented. **(F, G)** GSEA showing enrichment of gene sets in DMSO- or NHWD-870-treated 143B cell lines.

To further explore the molecular mechanism of NHWD-870 in osteosarcoma, we analyzed the gene expression profile of 143B and HOS osteosarcoma cell lines under NHWD-870 exposure (**Figure 2A**). The volcano plot of all the genes detected is shown in **Figure 2B**. Under our experimental conditions, a total of 904 downregulated genes (DEGs) (1833 genes were downregulated in 143B cells, 1518 genes were downregulated in HOS cells) were detected in two osteosarcoma cell lines exposed to 1 μM NHWD-870 compared with the DMSO control, using log2 fold change < -1 and adjusted p value < 0.01 as the threshold (**Figure 2C**). The overall downregulated DEG information was saved for further analyses.

Gene Ontology (GO) and Kyoto Encyclopedia of Genes and Genomes (KEGG) functional annotation of the identified DEGs was obtained using the DAVID online analysis tool (https://david.ncifcrf.gov/). The results were deemed statistically significant at the cutoff of FDR < 0.01, and the top 10 GO and KEGG terms of the downregulated DEGs are depicted in **Figures 2D, E**. The downregulated genes were mainly enriched in embryonic skeletal system development, the Rap1 signaling pathway, the NF-kappa B signaling pathway, and signaling pathways regulating the pluripotency of stem cells. Furthermore, gene set enrichment analysis (GSEA) was used to identify, characterize, and link potential biological pathways involved in the effects of NHWD-870 in osteosarcoma. Utilizing statistical cutoffs of a p value <0.05, we identified a series of tumor-proliferation-associated gene sets (focal adhesion, DNA replication and cell cycle) that were downregulated in the NHWD-870-treated group compared to the DMSO control group (**Figures 2F, G**).

The above results indicated that NHWD-870 reverses the oncogenic signature from oncogenic to tumor suppression. Among these pathways, the STAT3 signaling pathway (signaling pathways regulating the pluripotency of stem cells) acts mainly as a transcription activator in maintaining embryonic stem (ES) cell self-renewal and pluripotency by regulating downstream pluripotency target genes (11, 12), attracting our attention for further exploration.

### BRD4 Involves in JAK/STAT3 Activation Through Regulates the Upstream Receptor GP130

**Figure 3:**
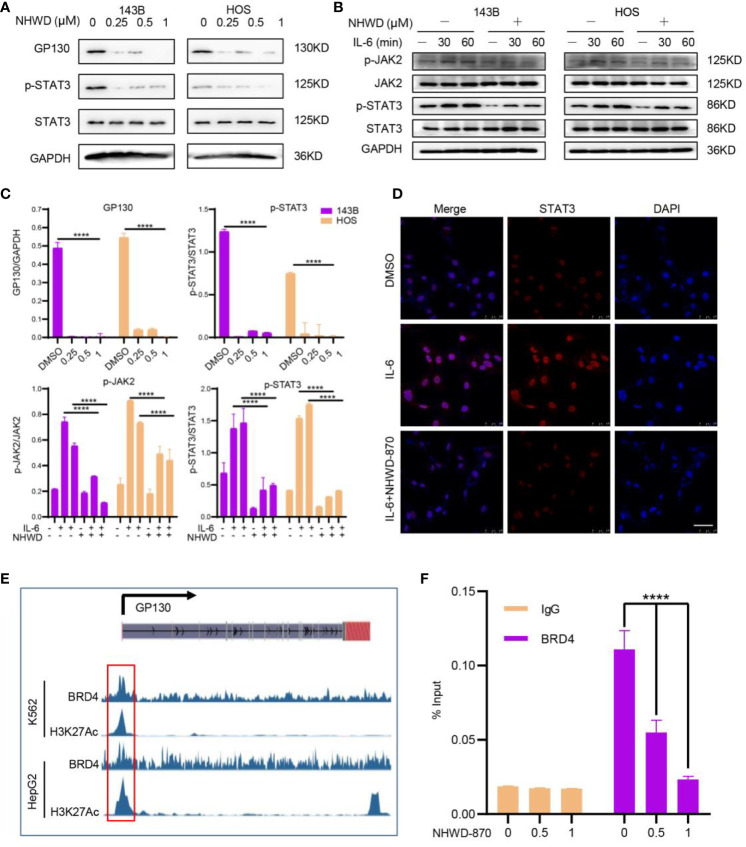
BRD4 regulates the activation of the JAK/STAT3 signaling pathway through GP130 receptor. **(A)** NHWD-870 directly inhibits the phosphorylation of STAT3 at tyrosine 705 in HOS and 143B osteosarcoma cell lines. **(B)** Under IL-6 stimulation, the STAT3 signaling pathway was significantly activated, and NHWD-870 effectively inhibited IL-6-induced STAT3 phosphorylation activation. **(C)** The expression levels of p-JAK and p-STAT3 were quantified using ImageJ software. (****P < 0.0001). **(D)** The nuclear translocation of STAT3 in 143B cells was determined using immunofluorescence. Scale bar = 50 μm. **(E)** The gene track for H3K27 acetylation and BRD4 in HepG2 and k562 cells from ENCODE. **(F)** ChIP-PCR for BRD4 association with the TSS of GP130 in 143B osteosarcoma cells treated with 1 µM NHWD-870 or DMSO using anti-BRD4 or anti-IgG as negative control. Data are expressed as the fold enrichment normalized to the input. (****P < 0.0001).

To further confirmed the effects of NHWD-870 on the activity of JAK/STAT3 signaling pathway, we first examined the expression level of JAK/STAT3 components under NHWD-870 treatment. Western blot results showed that the expression level of the STAT3 upstream receptor GP130 was significantly blocked by NHWD-870. Herein, we examined the impact of NHWD-870 on downstream effector molecules of the IL-6/GP130/STAT3 signaling pathway, including P-STAT3 (Y705) and P-JAK2. The results indicated that NHWD-870 blocked the phosphorylation of STAT3 at extremely low concentrations (**Figures 3A, C**). In addition, NHWD-870 significantly inhibited IL-6-induced JAK/STAT3 phosphorylation (**Figures 3B, C**). Immunofluorescence results showed that NHWD-870 significantly blocked the nuclear translocation of STAT3 induced by IL-6 (**Figure 3D**).

BRD4 largely acknowledged for its significant role in super-enhancers (SEs) organization and transcription regulation (13, 14). To exploring if BRD4 directly regulates GP130 expression through SEs, we first analyzed the distribution of H3K27Ac in the promoter region of GP130 in multiple cancer cell lines, including breast cancer (MCF-7), colon cancer (HCT116), Ewing sarcoma (A673), and osteosarcoma (SJSA1) et al. in ENCODE database (15). Consistently, tumor cells exhibits significant enhancer peak located in GP130 TSS region (**Supplementary Figure 2**). In MCF-7 and K562 cell line, H3K27Ac peak was largely in accordance with BRD4 (**Figure 3E**), indicating that BRD4 may regulates GP130 expression through super enhancers. This activation histone mark at the GP130 TSS was confirmed in 143B osteosarcoma cells by ChIP-PCR, while NHWD-870 abrogated the BRD4 enrichment at TSS site in a dose-dependent manner (**Figure 3F**).

Based on these results, we concluded that BRD4 directly regulates GP130 expression and the phosphorylation activation of the downstream JAK/STAT3 signaling pathway through binding to the TSS region.

### NHWD-870 Sensitizes Cisplatin by Repressing GP130

**Figure 4:**
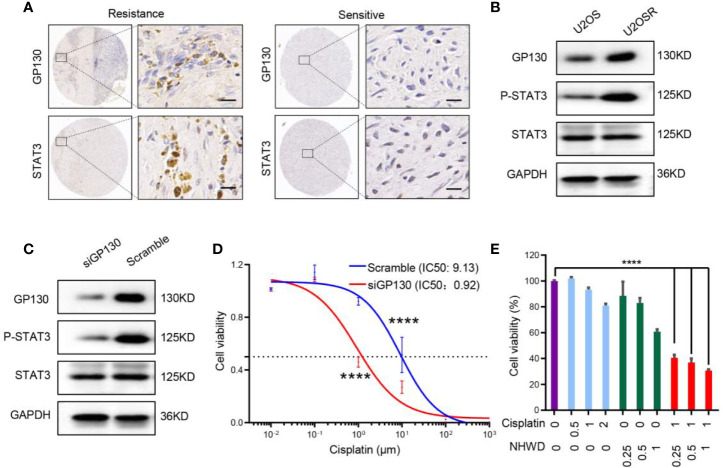
NHWD-870 sensitizes osteosarcoma cells to chemosensitivity by repressing GP130. **(A)** The expression level of GP130 in chemosensitive and chemoresistance patients was detected using immunohistochemistry. Scale bar = 50 μm. **(B)** The expression level of GP130 in chemosensitive (U2OS) and chemoresistance (U2OSR) osteosarcoma cell lines was detected using western blotting. **(C)** 143B cells were transfected with GP130 siRNA for 72 hours. GP130, p-STAT3 Y705, STAT3 and GAPDH were assessed by western blot. **(D)** Dose-response curve of the 143B scramble and 143B GP130 siRNA cell lines. Data are mean ± SEM. (****P < 0.0001). **(E)** Cells were treated with NHWD-870 and cisplatin individually or in combination at the indicated concentrations for 72 hours, and cell viability was assessed with a CCK8 assay. Data are shown as mean ± SD. (****P < 0.0001).

The JAK/STAT3 signaling pathway plays a critical role in tumor proliferation and differentiation, and it has been shown to confer chemoresistance (16, 17). To investigate the role of the upstream receptor GP130 in the acquisition of chemoresistance in osteosarcoma, we first analyzed the expression of GP130 in clinical samples as well as a pair of cell lines (U2OS and U2OSR) with different chemosensitivities (18). We found that GP130 was significantly overexpressed in chemo-resistance compared to chemo-sensitive osteosarcoma tissues (**Figure 4A**). In addition, western blot analysis showed that GP130 was highly expressed in chemo-resistance U2OSR cell line than the chemo-sensitive U2OS cell line (**Figure 4B**). Taken together, these results indicate that the upstream receptor GP130 may have important functions in osteosarcoma chemosensitivity regulation.

To reveal the relationship between GP130 expression and cisplatin sensitivity, we used small-interfering RNA targeting GP130, and the results showed that the activation of the STAT3 signaling pathway was significantly inhibited in the GP130 knockdown group (**Figure 4C**). More importantly, cell sensitivity to cisplatin was significantly increased after GP130 knockdown (**Figure 4D**). Since NHWD-870 could significantly inhibit GP130 expression, we speculated that NHWD-870 could increase cisplatin sensitivity. Indeed, our results showed that NHWD-870 worked synergistically with cisplatin in suppressing the viability of 143B osteosarcoma cell lines (**Figure 4E**). In conclusion, NHWD-870 increases cisplatin antitumor activation, which may be associated with blocking the GP130 receptor and the downstream signaling pathway.

### NHWD-870 Showed Robust Anti-Osteosarcoma Activity *In Vivo*

**Figure 5:**
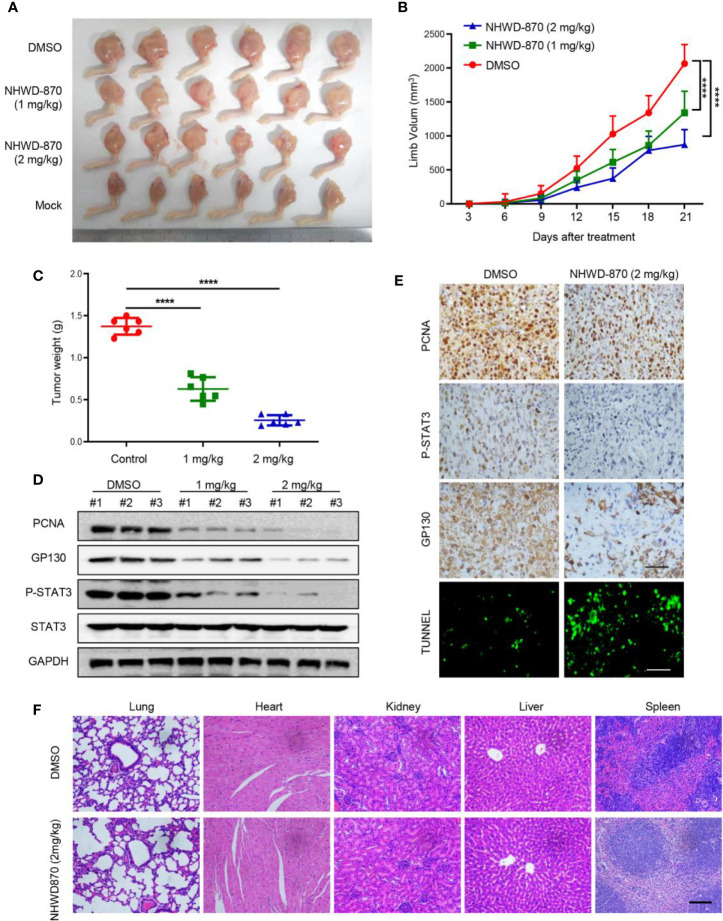
NHWD-870 inhibited the growth of osteosarcoma *in vivo*. **(A)** Gross tumor specimens in each group. **(B)** The daily change in tumor volume in each group and NHWD-870 showed dose-dependent inhibition of tumor growth *in vivo*. (****P < 0.0001). **(C)** Tumor weight of gross tumor specimens in each group at day 21, further demonstrating that NHWD-870 inhibited tumor growth. (****P < 0.0001). **(D)** Changes in the protein expression of PCNA, GP130 and activation of STAT3 signaling pathways were detected in tumor tissue in each group by western blotting. **(E)** Changes in the protein expression of PCNA, GP130 and activation of STAT3 signaling pathways in tumor tissues in each group were detected by immunohistochemistry, and TUNEL staining showed that NHWD-870 could significantly induce apoptosis in tumor tissues. Scale bar = 100 μm. **(F)** The systemic toxicity of NHWD-870 to vital organs *in vivo* was evaluated by H&E staining. Scale bar = 400 μm. (****, P < 0.0001).

To determine whether NHWD-870 inhibits osteosarcoma growth *in vivo*, 1 million 143B osteosarcoma cells was injected in the tibial to establish a tumor xenograft model (19). As the tumor was visible, the mice were randomly divided and treated with NHWD-870 at the indicated concentration (**Figure 5A**). The results indicated that NHWD-870 significantly inhibited tumor growth in a dose-dependent manner (**Figures 5B, C**). Western blotting and immunohistochemistry were adopted to further explore the molecular mechanism of NHWD-870 on tumor growth *in vivo*. The results indicated that NHWD-870 significantly inhibited GP130, PCNA and the phosphorylation of STAT3 (Tyr705) in the experimental group (**Figures 5D, E**). TUNEL analyses also showed that NHWD-870 promote cell apoptosis *in vivo* (**Figure 5E**). H&E staining indicated that NHWD-870 had no significant toxic effects on vital organs (**Figure 5F**). In summary, NHWD-870 effectively inhibit the growth of osteosarcoma by targeting STAT3 *in vivo*.

### NHWD-870 Attenuates OS-Induced Bone Destruction by Inhibiting Osteoclast Differentiation

The homeostasis of bone structure depends on the dynamic balance of osteoblasts and osteoclasts. As the unique cell type in bone resorption, osteoclasts play an important role in osteolytic disease (20, 21). Activation signaling in osteoclasts, including the NF-κB signaling pathway ligand (RANKL), tumor necrosis factor (TNF) and tumor necrosis factor receptor family, TNFSF11, TNFRSF11A, and TNFRSF11B, is a key regulatory factor in bone metabolism (22, 23). Osteoclasts perform the function of bone absorption and provide nutrition and a suitable microenvironment for tumor cell proliferation. Osteoclasts and tumor cells support each other, forming a vicious cycle and accelerating the progression of both bone tumors and bone destruction (24, 25).

**Figure 6:**
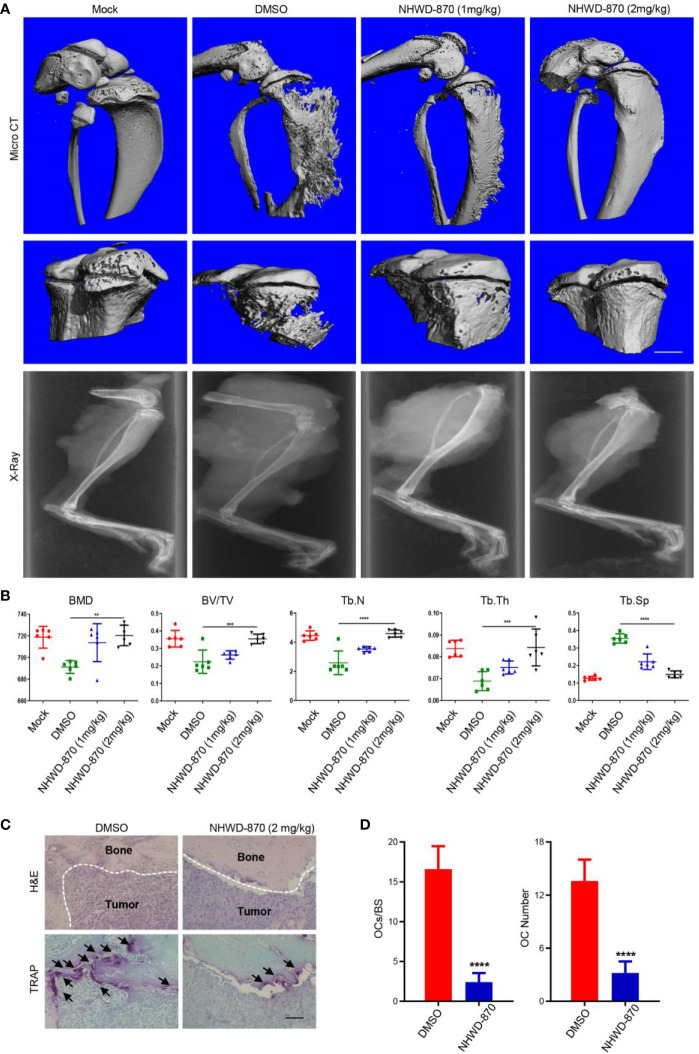
NHWD-870 inhibits tumor-induced osteolysis by inhibiting the differentiation and maturation of osteoclast precursor. **(A)** The effect of NHWD-870 on inhibiting tumor-induced osteolysis *in vivo* in each group was detected by micro-CT. Scale bar = 1 mm. **(B)** Relative parameters of the bone microstructure in each group. (**P < 0.01; and ***P < 0.001, and ****P < 0.0001). **(C)** The number and distribution of osteoclasts at the tumor-to-normal bone boundary was detected by TRAP. Scale bar = 100 μm. **(D)** A significant decrease in the number and distribution of osteoclasts demonstrated that NHWD-870 inhibited the differentiation and maturation of osteoclasts, preventing tumor-induced osteolysis. (****P < 0.0001).

In this study, we found that the bone destruction of mice treated with DMSO was more serious than that of the NHWD-870 group (**Figure 6A**). Microstructural analysis of the tibia indicated that NHWD-870 effectively maintained the bone mass of mice and slowed the bone destruction effects induced by bone tumors (**Figure 6B**). TRAP staining was used to detect the number and distribution of osteoclasts at the interface between the tumor and bone, and the results showed that the number and distribution of osteoclasts in the NHWD-870-treated group were significantly lower than those in the control group (**Figures 6C, D**). On this basis, we used mouse bone marrow-derived macrophages (BMM) cells to explore the effects of NHWD-870 on osteoclast precursor differentiation *in vitro*. The results indicated that under RANKL stimulation, BMM cells could differentiate into polynuclear giant cells with the integrity of the actin skeleton, and this process could be significantly reversed by a low dose of NHWD-870 (**Supplementary Figure 3**). Combined with the above results, NHWD-870 reduced osteoclast-mediated bone resorption in osteosarcoma by inhibiting osteoclast differentiation and maturation.

### NHWD-870 Induced Synthetic Lethality to Cisplatin Chemotherapy in a Preclinical Osteosarcoma PDX Model

The PDX model is considered to provide a powerful tool for translational research as it retains the key molecular and biological characteristics of the corresponding primary tumor (26, 27). Moreover, the PDX model was proven to have ideal biological stability, which can ensure that the genetic information can be effectively retained during passage. Therefore, compared with rare clinical samples, the PDX model can provide more stable and lasting genetic information for molecular biology research (28).

**Figure 7:**
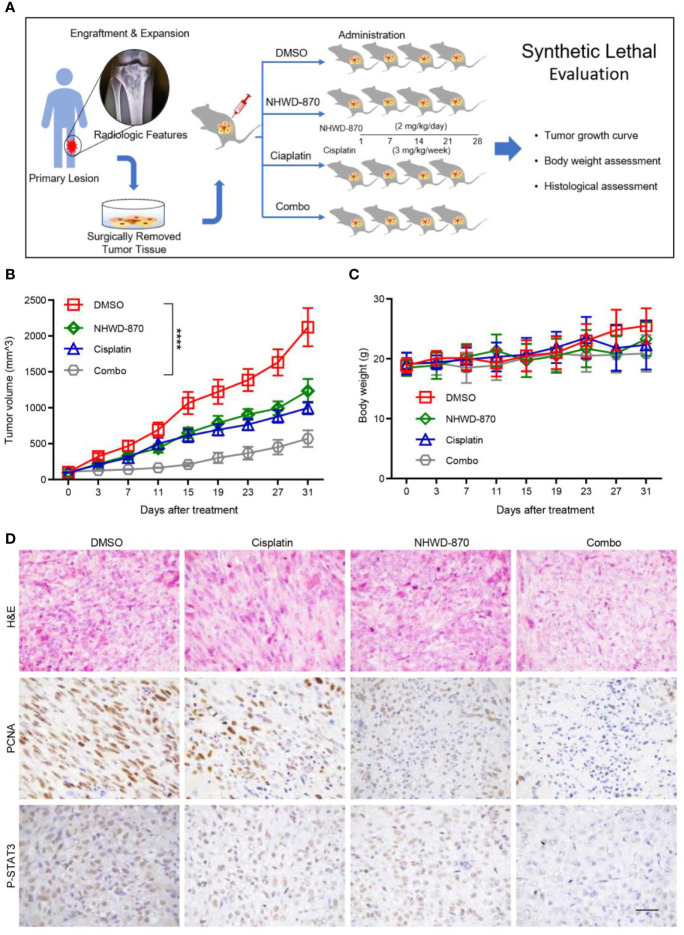
NHWD-870 sensitizes cells to cisplatin in a preclinical osteosarcoma PDX model. **(A)** Flowchart of our *in vivo* drug validation study. **(B)** Daily changes in tumor volume demonstrated that the combination of NHWD-870 and cisplatin enhanced the inhibition of osteosarcoma *in vivo*. (****P < 0.0001). **(C)** Daily change in the body weight of the PDX model in each group. **(D)** Histological malignancy reduction of osteosarcoma tissue under the enhanced inhibition of the drug combination was detected by H&E, and the relative decreased activation of proliferation-associated antigens and STAT3 was detected by immunohistochemistry. Scale bar = 50 μm.

In our study, we successfully constructed a PDX model using tumor tissue from a patient with recurrent osteosarcoma. The model can be stably passed on in nude mice. The third-generation tumor model was selected for the experiments. As tumor growth reached 100 mm^3^, the model was randomly assigned into 4 groups with 4 mice in each group. Mice in each group were treated with NHWD-870 or cisplatin alone or in combination to evaluate the synergistic anticancer effects of NHWD-870 and cisplatin in the osteosarcoma model (**Figure 7A**). The results showed that NHWD-870 and cisplatin alone inhibited the growth of PDX model tumors to a certain extent, and more importantly, the combination of NHWD-870 and cisplatin resulted in a significantly greater reduction in tumor growth than either of them individually without significant side effects on body weight (**Figures 7B, C**). Immunohistochemical analysis of tumor tissues showed that NHWD-870 combined with cisplatin more effectively inhibited the expression of the tumor proliferative antigen PCNA. In addition, the inhibition of the STAT3 signaling pathway was more pronounced in the combination group (**Figure 7D**). These data suggest that NHWD-870 applied in preclinical cancer models effectively inhibits the growth of osteosarcoma. Moreover, NHWD-870 enhances the antitumor effects of conventional chemotherapy. The favorable therapeutic properties of NHWD-870 demonstrate the potential of this compound as a potent therapeutic agent for cancer treatment in the future.

## Discussion

Osteosarcoma is the most common primary malignant bone tumor in children and adolescents with high metastatic and fatal rate. The 5-year survival rate of osteosarcoma is approximately 70% (29). However, the 5-year survival rate is only approximately 20% in metastatic patients (30). Therefore, further in-depth study focus on the molecular mechanism of chemotherapy resistance and metastasis is urgently needed.

**Figure 8:**
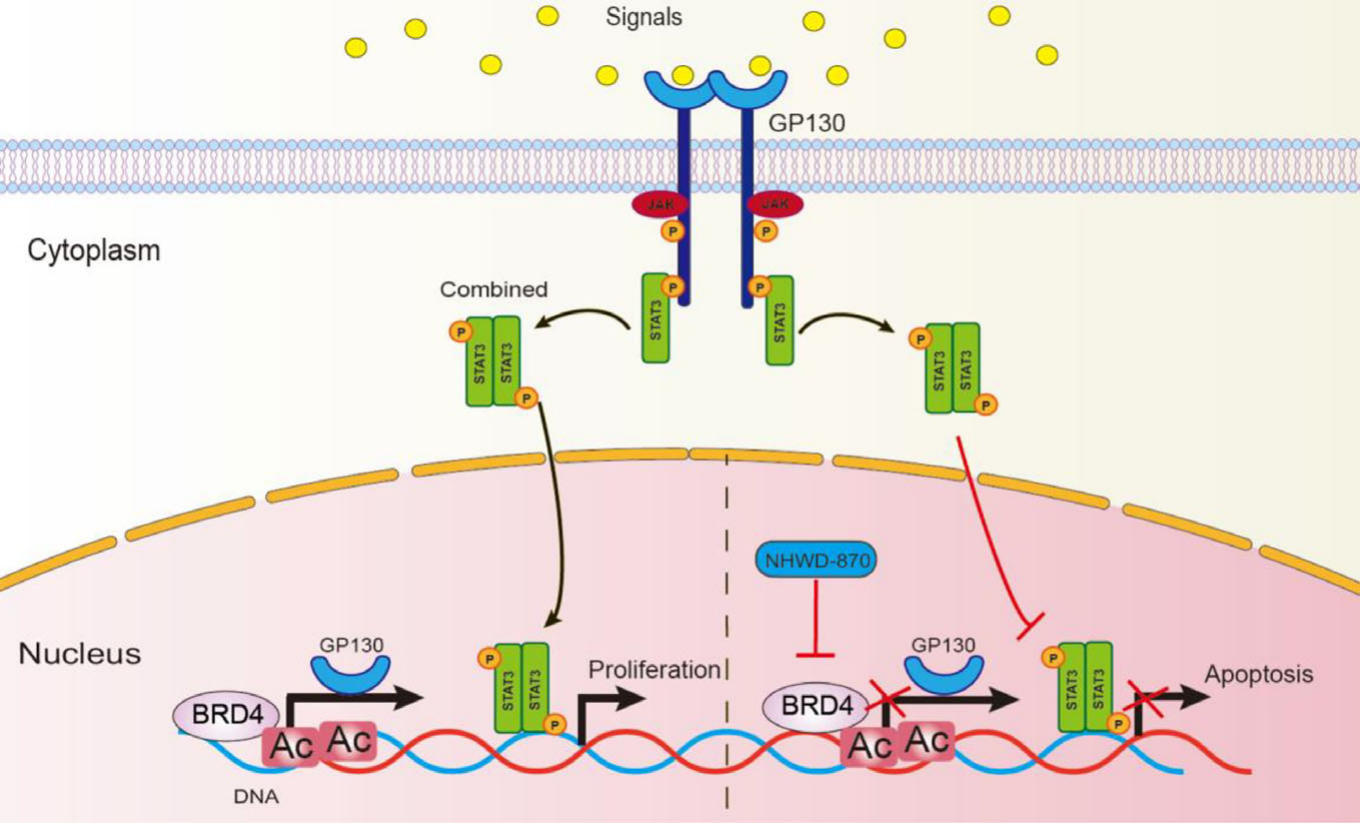
Schematic diagram to describe the action of NHWD-870. STAT3 is phosphorylated by JAKs at tyrosine 705, leading to STAT3 dimerization and nuclear translocation, followed by STAT3-mediated transcription of target genes and cell proliferation. In this process, NHWD-870 disrupted the binding affinity of BRD4 to the promoter region of STAT3 upstream receptor GP130, this weakens the upstream signal transduction, blocking STAT3 dimerization and induced cell apoptosis.

The ability of BET inhibitor targeting oncogenic pathways in different cancers endows its extensive clinical applications, and increasing number of FDA-approved BET inhibitors have been studied in variety of cancers (31–33). In our previous studies, we explored the ability of a potent BRD4 inhibitor NHWD-870 shows great tumor suppressive potential in multiple solid tumors by depleting phosphorylated BRD4 and c-MYC (8, 9). In the current study, we further explored the anti-osteosarcoma activity of NHWD-870 and the related mechanisms. NHWD-870 reduced the proliferation and invasion of osteosarcoma cells by reversing the oncogenic signature *in vitro*. To our surprise, NHWD-870 showed potent inhibitory effects on JAK/STAT3 signaling pathway activation and BRD4 was identified as a direct regulator of JAK/STAT3. NHWD-870 disrupt the binding affinity of BRD4 to the promotor of upstream receptor GP130, and further inhibited JAK/STAT3 signaling pathway activation (**Figure 8**).

The activation of the STAT3 signaling pathway was identified as a major factor involved in the occurrence of osteosarcoma and the regulation of tumor proliferation and metastasis (10, 34). Newly identified regulators and functions of JAK-STAT3 was of great significance in osteosarcoma therapy (35). In our study, GP130 knockdown enhances chemotherapy upon cisplatin treatment *in vitro*. In addition, NHWD-870 increases the antitumor activity of cisplatin, which may be associated with blocking the GP130 receptor and the downstream signaling pathway.

Osteosarcoma is a bone-derived tumor, and its unique microenvironment inevitably induces and stimulates osteoclast-mediated bone resorption (24). There are two different pathologic types of bone tumors—osteolytic and osteoblastic (36). Osteoclasts play an important role in the progression of both types of pathologic types. In osteolytic lesions, bone destruction is mediated by osteoclasts rather than by the direct action of cancer cells (37). Tumor cells produce many factors that stimulate osteolysis, such as PTHrP, IL-1, IL-6, IL-8, IL-11, and transforming growth factors, which directly inducing RANKL-mediated osteoclast differentiation and maturation (38). This vicious cycle means that the therapeutic strategies for osteoclast differentiation and regulation processes are of great significance. In our study, NHWD-870 directly inhibited osteoclast differentiation and maturation. In addition, NHWD-870 significantly delayed bone destruction in an orthotopic xenograft model *in vivo*. For this reason, pharmacological inhibition of JAK/STAT3 with NHWD-870 appears to be a promising strategy for the treatment of bone-associated tumors, as it disturbs the vicious cycle between tumor progression and bone remodeling.

PDX models directly transferred the patient’s tumor tissue into immunodeficient mice, which simulated the characteristics of the primary tumor and retained tumor heterogeneity (26). We found that NHWD-870 significantly inhibited tumor growth and synergy with cisplatin. In addition, NHWD-870 significantly inhibited the phosphorylation of STAT3 *in vivo*. These results further highlight the clinical translational potential of NHWD-870.

In conclusion, pharmacological inhibition of BET appears to be a promising strategy for the treatment of osteosarcoma, as it interferes with the vicious cycle between bone tumor progression and bone destruction. As a potent compound to simultaneously inhibit bone tumors as well as osteoclast differentiation associated with low toxicity, NHWD-870 is thought to be a promising therapeutic agent for future development of antineoplastic drugs.

## Data Availability Statement

The datasets (gene.count.matrix) presented in this study can be found in **Supplementary Material**.

## Ethics Statement

The studies involving human participants were reviewed and approved by Ethics Committee of Shanghai General Hospital. Written informed consent to participate in this study was provided by the participants’ legal guardian/next of kin. The animal study was reviewed and approved by Animal Ethics Committee of Shanghai General Hospital.

## Author Contributions

YH, WS, MZY, and ZC conceived of the presented idea and researched the background of the study. YJ, WG, and HM prepared the figures and tables. YL, XM, JX, and ZW established the PDX model. JW and YQL analyzed genomic and transcriptome sequencing data. JH, ZW, KZ, and MY performed cell biology experiments. TZ, SX, XJ, and YJ performed animal experiments. YJ and HM wrote the manuscript. All authors contributed to the article and approved the submitted version.

## Funding

This study was supported by National Natural Science Foundation of China (81972512, 81872177, 81772859, 82072969 and 82072968). the Shanghai Science and Technology Commission (14140904000), Shanghai Sailing Program (NO.19YF1440200) and Shanghai Jiaotong University Translation Medicine Cross Research Fund (ZH2018QNA17), and the Shanghai Hospital Development Center (SHDC2020CR2037B).

## Conflict of Interest

The authors declare that the research was conducted in the absence of any commercial or financial relationships that could be construed as a potential conflict of interest.
