# Supplementary material for: Bromodomain Inhibition Attenuates the Progression and Sensitizes the Chemosensitivity of Osteosarcoma by Repressing GP130/STAT3 Signaling
Source: Front Oncol. 2021 Jun 8;11:642134. doi: 10.3389/fonc.2021.642134 (PMC8219214; doi:10.3389/fonc.2021.642134)

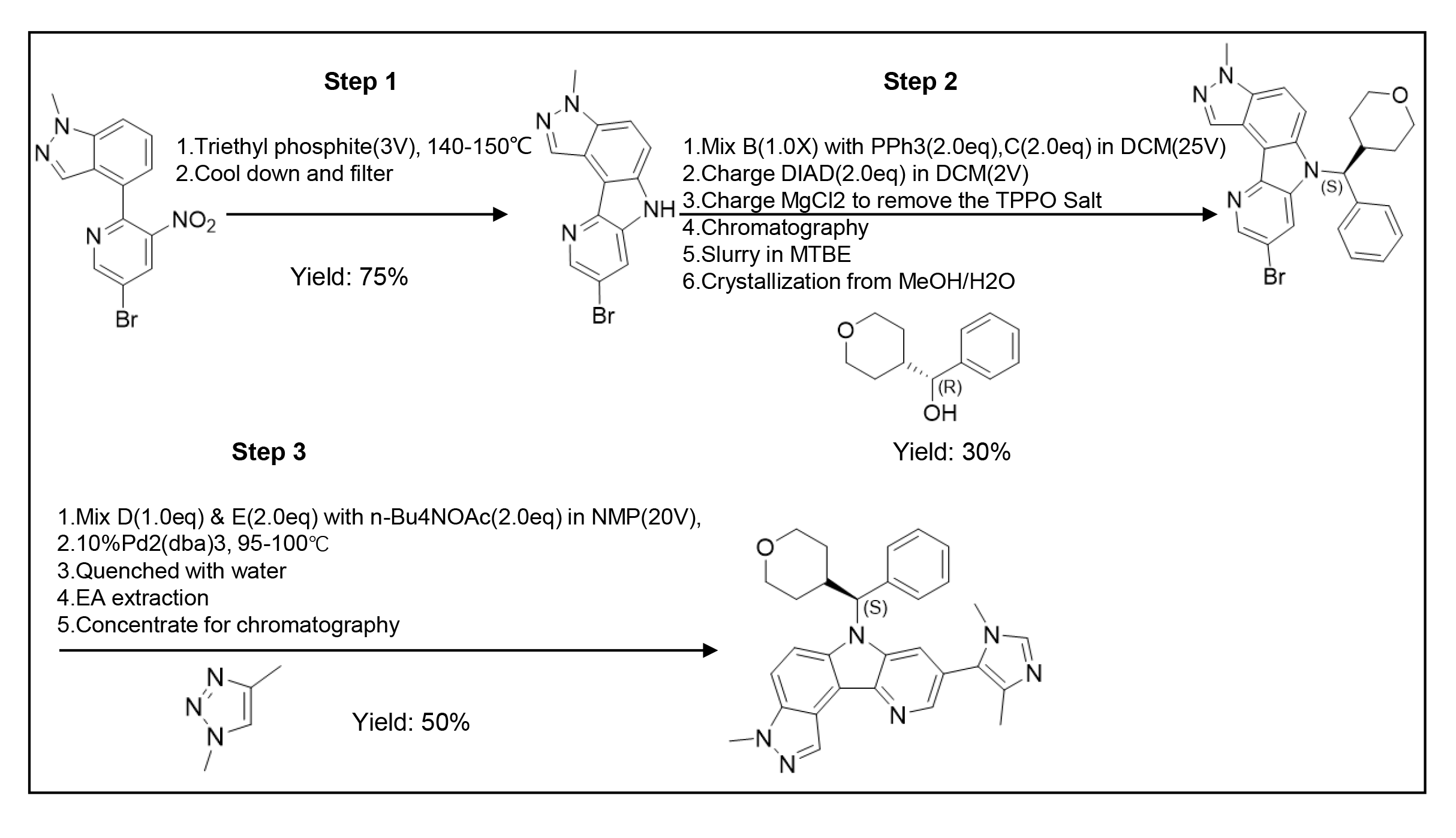
**Supplementary figure 1. The synthesis process of NHWD-870.**

**Supplementary figure 2. The gene track for H3K27 acetylation in multiple tumor cell lines from ENCODE.** Neuroblastoma (SK-N-SH), Breast cancer (MCF-7), Acute promyelocytic leukemia (APL, HL60), Lung cancer (PC-9), Non-Hodgkin lymphoma (NHL, OCI-LY3), Colon cancer (HCT116), Ewing sarcoma (A673), Pancreatic cancer (PANC1), Prostate cancer (22RV1), Myeloma (MM.1S), and a osteosarcoma cell line SJSA1.

**
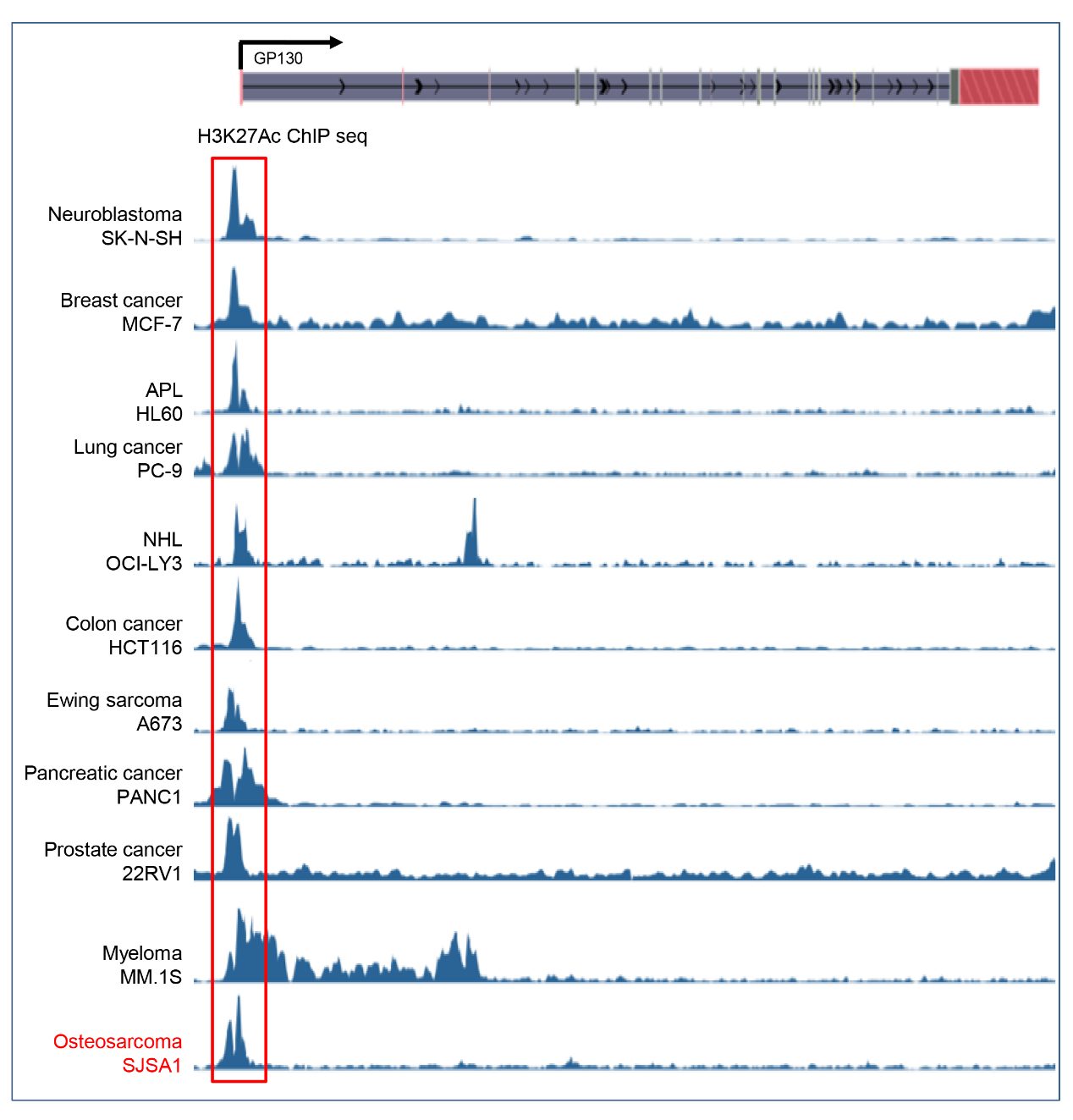
**

**Supplementary figure 3. NHWD-870 reduced RANKL-mediated osteoclast differentiation.** The formation of F-actin ring in BMMs stimulated with RANKL in presence and absence of NHWD-870. Scale bar = 200 μm.


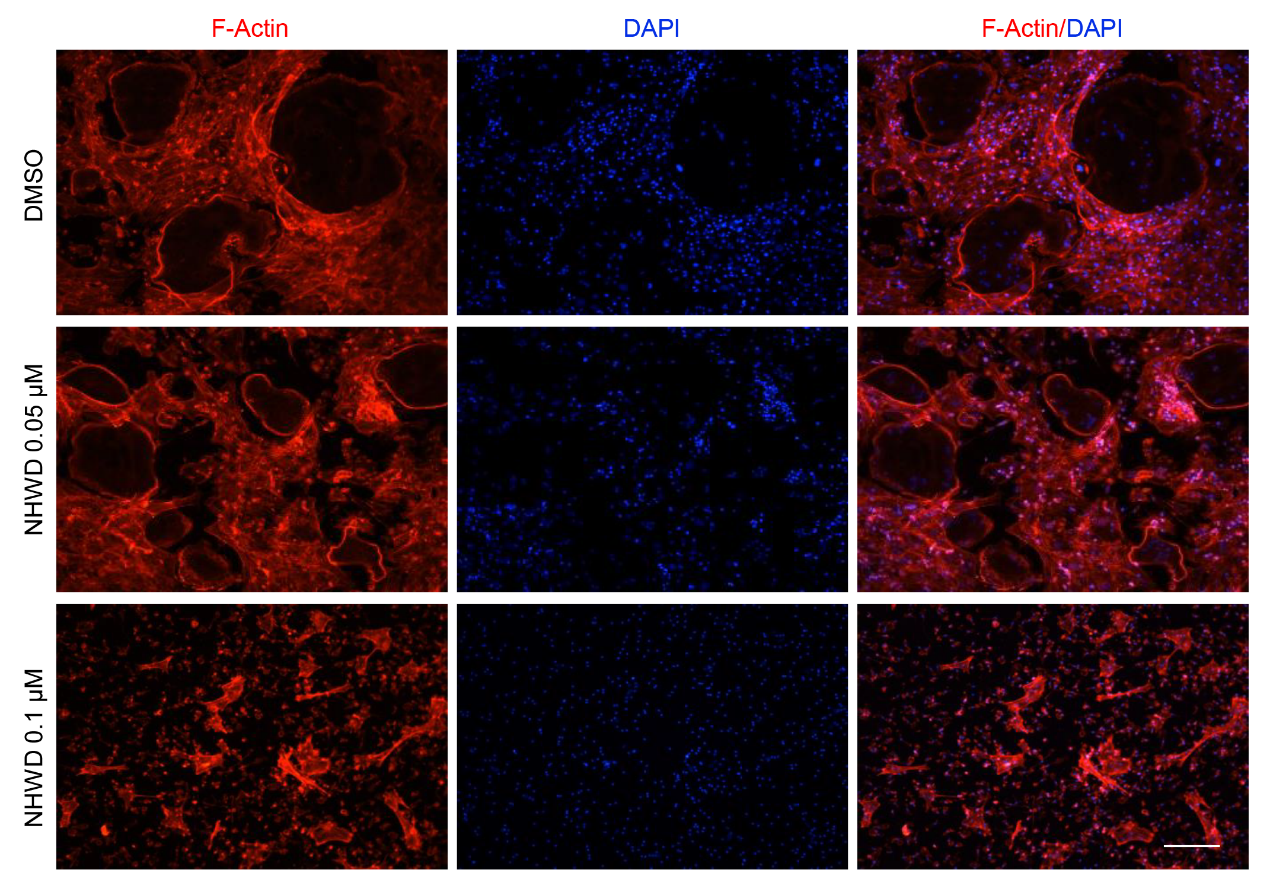

Supplement: Supplementary file 1 [file DataSheet_1.docx]
